# Supplementary material for: Robust and generalizable embryo selection based on artificial intelligence and time-lapse image sequences
Source: PLoS One. 2022 Feb 2;17(2):e0262661. doi: 10.1371/journal.pone.0262661 (PMC8809568; doi:10.1371/journal.pone.0262661)
Supplement: S1 Fig — Density plots of the FH+ (green), FH- (blue) and discarded (red) embryos evaluated on a single validation fold for the three different sampling strategies. The left panel shows the distribution of the raw sigmoid output from a model that was trained only on KID embryos. In the mid panel a model was trained only on KID embryos but with oversampling of FH+ embryos. In the right panel a model was trained using oversampling of FH+ and including discarded embryos. (PDF) [file pone.0262661.s001.pdf]

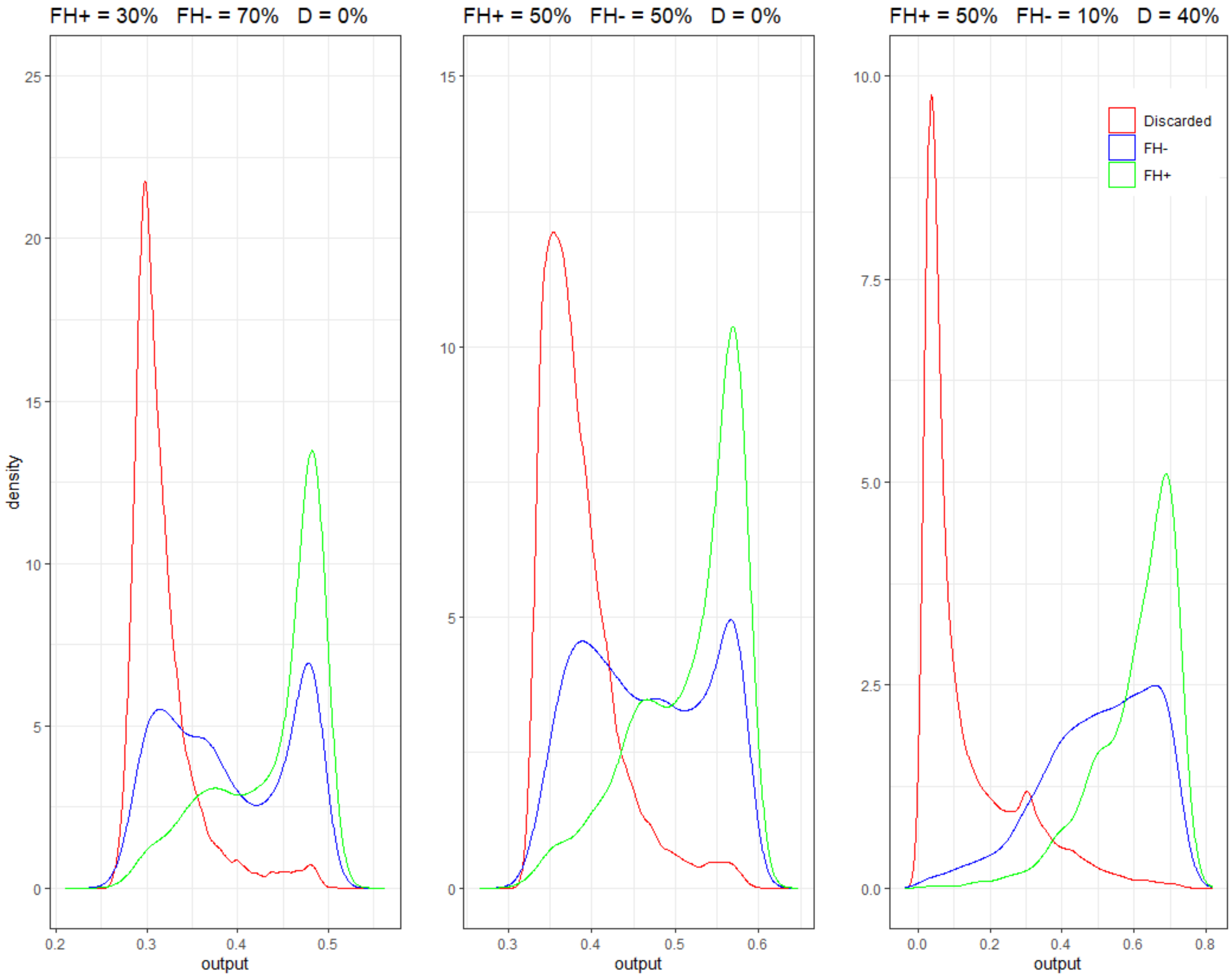

Supplementary Figure 1. Density plots of the FH+ (green), FH- (blue) and discarded (red) embryos evaluated on a single validation fold for the three different sampling strategies. The left panel shows the distribution of the raw sigmoid output from a model that was trained only on KID embryos. In the mid panel a model was trained only on KID embryos but with oversampling of FH+ embryos. In the right panel a model was trained using oversampling of FH+ and including discarded embryos.
