# Supplementary material for: Robust and generalizable embryo selection based on artificial intelligence and time-lapse image sequences
Source: PLoS One. 2022 Feb 2;17(2):e0262661. doi: 10.1371/journal.pone.0262661 (PMC8809568; doi:10.1371/journal.pone.0262661)
Supplement: S2 Fig — Precision/recall curve for KID embryos in the test data set (n = 2,212) based on iDAScore predictions. The dotted line shows the prevalence of FH+ in the data set. (PDF) [file pone.0262661.s002.pdf]

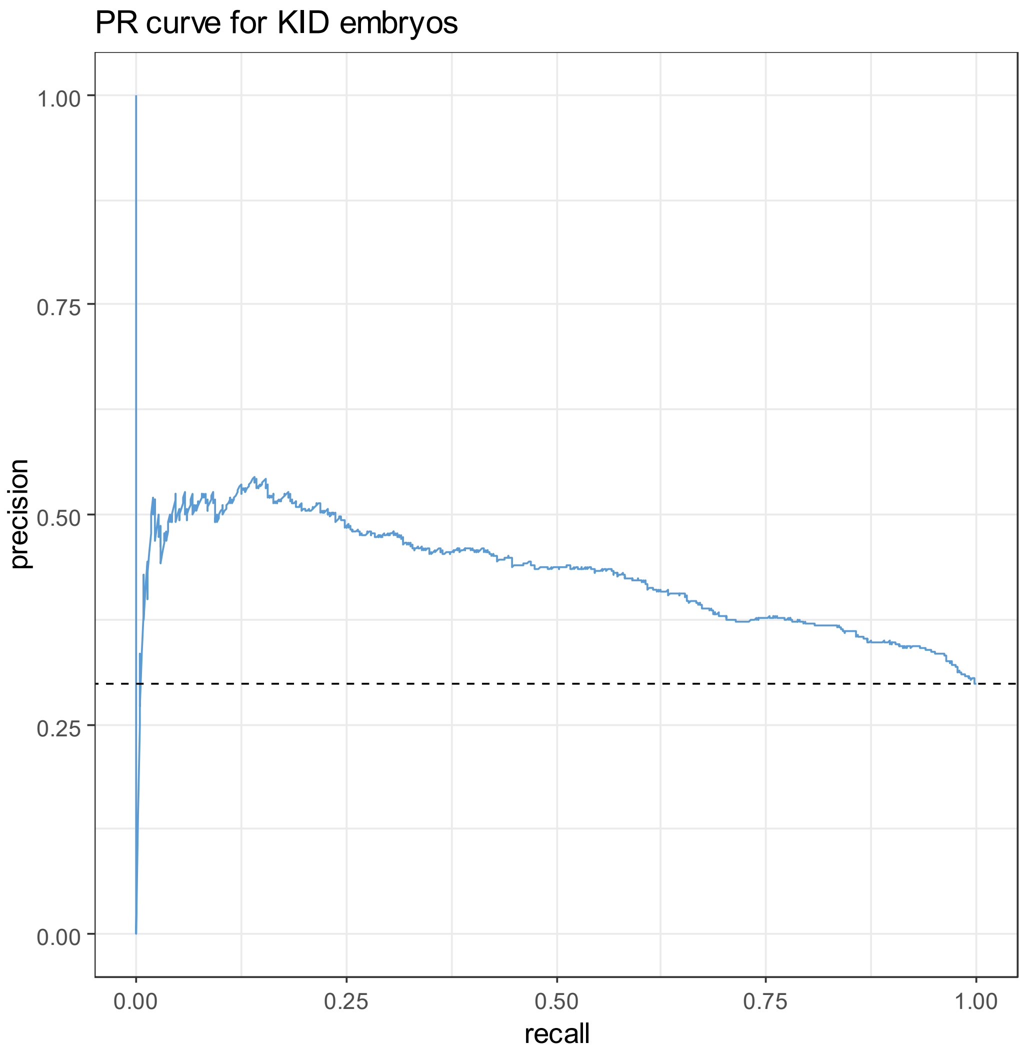

Supplementary Figure 2. Precision/recall curve for KID embryos in the test data set (n = 2,212) based on iDAScore predictions. The dotted line shows the prevalence of FH+ in the data set.
